# Mobile-Based and Self-Service Tool (iPed) to Collect, Manage, and Visualize Pedigree Data: Development Study

**DOI:** 10.2196/36914

**Published:** 2022-06-23

**Authors:** Chen Sun, Jing Xu, Junxian Tao, Yu Dong, Haiyan Chen, Zhe Jia, Yingnan Ma, Mingming Zhang, Siyu Wei, Guoping Tang, Hongchao Lyu, Yongshuai Jiang

**Affiliations:** 1 College of Bioinformatics Science and Technology Harbin Medical University Harbin China; 2 The Fourth Affiliated Hospital Zhejiang University School of Medicine Zhejiang China

**Keywords:** pedigree, pedigree data, visualization, self-service, mobile-based

## Abstract

**Background:**

Pedigree data (family history) are indispensable for genetics studies and the assessment of individuals' disease susceptibility. With the popularity of genetics testing, the collection of pedigree data is becoming more common. However, it can be time-consuming, laborious, and tedious for clinicians to investigate all pedigree data for each patient. A self-service robot could inquire about patients' family history in place of professional clinicians or genetic counselors.

**Objective:**

The aim of this study was to develop a mobile-based and self-service tool to collect and visualize pedigree data, not only for professionals but also for those who know little about genetics.

**Methods:**

There are 4 main aspects in the iPed construction, including interface building, data processing, data storage, and data visualization. The user interface was built using HTML, JavaScript libraries, and Cascading Style Sheets (version 3; Daniel Eden). Processing of the submitted data is carried out by PHP programming language. MySQL is used to document and manage the pedigree data. PHP calls the R script to accomplish the visualization.

**Results:**

iPed is freely available to all users through the iPed website. No software is required to be installed, no pedigree files need to be prepared, and no knowledge of genetics or programs is required. The users can easily complete their pedigree data collection and visualization on their own and through a dialogue with iPed. Meanwhile, iPed provides a database that stores all users’ information. Therefore, when the users need to construct new pedigree trees for other genetic traits or modify the pedigree trees that have already been created, unnecessary duplication of operations can be avoided.

**Conclusions:**

iPed is a mobile-based and self-service tool that could be used by both professionals and nonprofessionals at any time and from any place. It reduces the amount of time required to collect, manage, and visualize pedigree data.

## Introduction

Genetics plays a vital role in all diseases, and therefore, pedigree data are frequently used for clinical diagnosis [1]. Over the past few years, more attention has been paid to formulating an efficient model for disease risk prediction. Accurate risk prediction can be of significant help in disease prevention and follow-up strategies [2]. There is consensus that family history is an important risk factor in estimating an individual’s susceptibility to diseases, especially hereditary diseases, as it reflects the shared heredity and environment [3-5]. Numerous studies have applied family history as a risk factor to study the treatment and outcomes of schizophrenia [6,7]; predict cardiovascular disease risk [8]; and assess the risk of common cancers such as gastric cancer [9], colorectal cancer [10], and breast cancer [11]. However, pedigree data collection is so limited by familiarity and family communication of patients that it is hard to acquire in a clinical environment [12]. In addition, genetic information will change over time as the number of family members and confirmed relatives increases [12].

To address these issues, several web-based tools have been developed to collect and visualize pedigree data. However, it is indispensable for clinicians and genetics counselors to possess professional knowledge in order to use these tools. For example, *Pedigreejs* [13] is a pedigree editor based on JavaScript that can produce SVG format images in web browsers [14]; however, it is not easy to use for many users who are unfamiliar with JavaScript or specific web programming visualization libraries [15]. *Ped_draw* [15] can generate an image file as a command line or web tool on the condition that users prepare pedigree data first. *Panogram* [16] and *HaploPainter* [17] are both software to visualize the pedigree data, while the image file is not free to use.

We developed iPed, a mobile-based and self-service tool, to collect and visualize pedigree data. Mobile-based technologies are more widespread than web-based technologies, and they can improve the quality of life and reduce health care costs effectively [18,19]. Collection and visualization of pedigree data is accomplished by responding to some questions posed by iPed. There is no need to prepare an input file or perform any other operation except tapping on some options on the phone. iPed’s intelligent robot significantly reduces the work pressure of the clinician in querying about the patient’s family history. Furthermore, by storing pedigree data in a database, iPed makes it more convenient for users to manage their information.

## Methods

### Overview of Modules

iPed has 3 main parts: (1) *Your pedigree*, which stores information about family members; (2) *History*, which includes the phenotypic information users have previously entered and the visualizations; (3) *New*, which collects family information about a selected or newly created phenotype through answers to some questions asked by iPed. The interface was built using HTML, which includes a set of tags that unify the format of documents on the network and connect scattered internet resources into a logical whole. JavaScript libraries are used to respond to browser events, including changing information of family members in *Your pedigree*, modifying the phenotype data in *History*, and the dialogue with iPed when creating a new phenotype in *New*. The combination of HTML and JavaScript can complete the interaction between users and iPed. In addition, Cascading Style Sheets (version 3; Daniel Eden), a language that defines style structures such as font, color, and position, is applied to modify web pages.

### Data Processing

ThinkPHP (version 3.1.3; Chen Liu et al), a fast, compatible, and simple lightweight PHP development framework, is applied for the processing of the submitted data. The user could tap on the options to answer questions asked by iPed. When the dialogue ends, the data collection is finished at the same time. iPed will then convert the data into a specific format for further storage and visualization.

### Data Storage

All data including family members’ age and phenotypic information are stored in MySQL (version 5.0; Oracle Corporation), the most commonly used relational database management system. PhpMyAdmin (version 3.3.7; The phpMyAdmin Project) is a PHP and web-based MySQL database management tool for managing MySQL databases with a web interface.

### Pedigree Data Visualization

iPed completes the data visualization by calling the R script in PHP. Kinship2 [20], an R package restructured from kinship package [21], is used to visualize the pedigree data. Conventionally, squares and circles represent males and females, respectively. If the person has the phenotype, the pattern will be colored black; otherwise, it will be white. The connecting lines between patterns indicate genetic relationships.

### Ethics Consideration

This study did not require ethics approval as it did not involve any human subjects and the mobile-based tool was developed for visualization of pedigree data for all users.

## Results

### Pedigree and First Phenotype Data Collection

**Figure 1:**
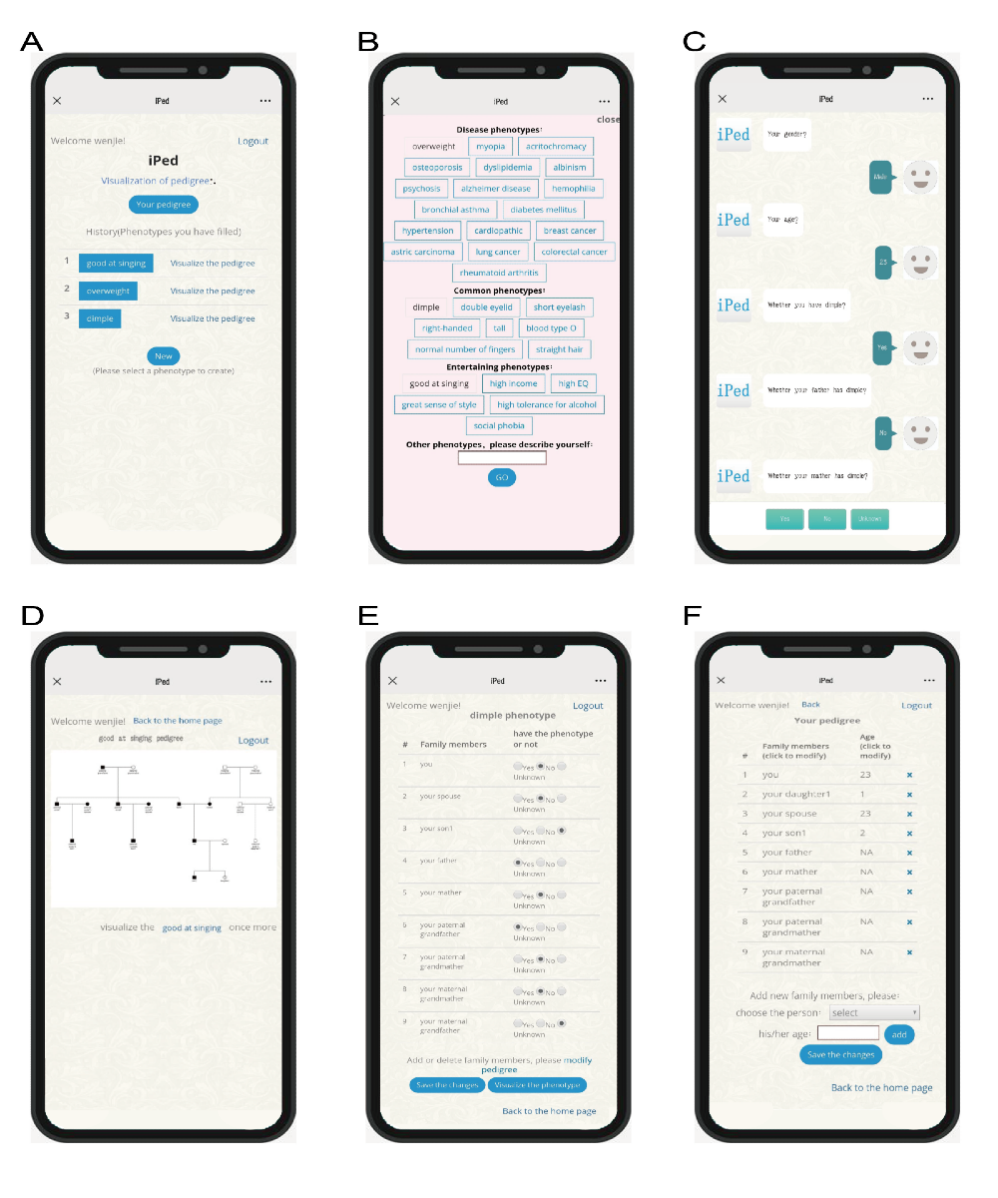
Main pages when the user logs back into iPed the next time. (A) The home page; (B) alternative phenotypes on iPed when the user taps on *New*; (C) the dialogue between the user and the robot when the user needs to define a new phenotype; (D) visualization of the genetic phenotypic data; (E) the genetic phenotype the user has entered, including the family members and their phenotypic information; (F) *Your pedigree* shows the family members and their ages.

**Figure 2:**
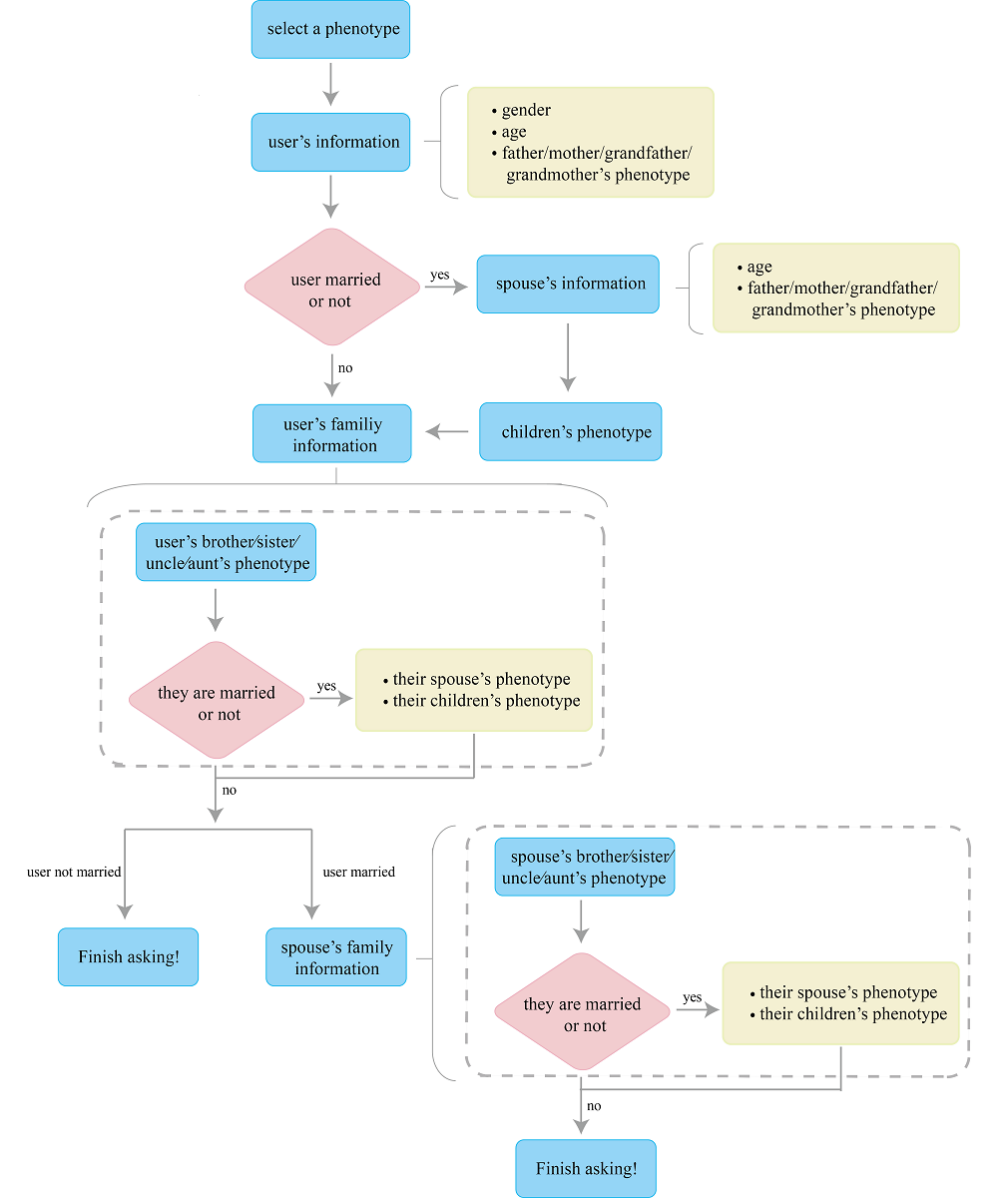
The flow path of the questions asked by the intelligent robot when the user logs into iPed for the first time.

iPed is a mobile-based, web-based, self-service tool for collecting and visualizing pedigree data on genetic phenotypes. A first-time user needs to register to save personal information for the next use. After a successful login, the user can select a phenotype of interest (Figure 1B), including disease phenotypes (eg, lung cancer, breast cancer, and rheumatoid arthritis), common phenotypes (eg, double eyelid, tall, and right-handed), and entertaining phenotypes (eg, singing well, high income, or social phobias). The user can also create a new phenotype and complete the family data collection for the created phenotype through an interactive dialogue with an intelligent robot (Figure 1C). The robot will ask some questions about the user’s age, whether the user has the phenotype, whether the user has a spouse or child, and whether the user’s relatives and their families have the phenotype (Figure 2).

After the first use, iPed will save the user’s family information in *Your* *Pedigree* (Figure 1F), including family members and their ages. This makes it convenient to directly collect pedigree data about the user’s family members when the user describes a new genetic phenotype.

When the users log in again, they can check their family information (*Your pedigree*), view the genetic phenotypic information (eg, the phenotype name, such as *dimple*), and the corresponding visualization (*Visualize the pedigree*). They can also select a new phenotype on the home page (*New*) to visualize the information (Figure 1A).

### Phenotype Data Visualization and Modification

#### Phenotype Data Visualization

Phenotype data visualization is accessible by tapping on *Visualize the pedigree*, and the user can see the visualized phenotypic information on the result page (Figure 1D). When the users find information that needs to be changed, they can tap on *Visualize the phenotype once more* to complete the modification.

#### Phenotype Data Modification

When information needs to be modified, the user can just click on the name of phenotype such as *dimple* (Figure 1A) or tap on *Visualize the phenotype once more* on the result page (Figure 1D) and change the targeted family member’s status on the page showing phenotypic information (Figure 1E). If the family member’s status is changed, tapping on *Modify pedigree* (Figure 1E) will direct the user to *Your pedigree*. *Your* *Pedigree* provides options to add or delete family members and modify their ages. It should be noted that the already existing family members cannot be added again. After modifying the family member’s information, the user can return to change the member’s phenotypic information. After finishing all the modifications, the user can visualize the phenotypic information afresh.

### New Phenotype Data Adding

If users intend to visualize new phenotypic data, they could tap on *New* on the home page, select an unfulfilled phenotype that they are interested in (Figure 1B), and complete the dialogue with the robot. The robot will simply ask whether the members added by the user have this phenotype. Afterward, it will generate the corresponding visualization.

## Discussion

### Principal Findings

Pedigree data are crucial in genetics studies and disease diagnosis, development, and prognosis. There are thousands of studies that have applied family history as a risk factor. However, this information is not easy to collect and manage. iPed provides a self-service tool for users to collect and visualize this information. With iPed, users do not need to prepare an input file first or learn any knowledge about genetics. They can visualize their pedigree data just by answering the intelligent robot’s questions, without any professional operations. At the same time, all the information submitted by users is stored in a database, so that visualizing a new phenotype or modifying previously entered data is more convenient.

### Comparison With Prior Work

Recently, some web-based tools for collecting and visualizing pedigree data have been developed. There are some limitations to these existing tools; if the user would like to use these tools, they have to gain some knowledge first. For example, *Pedigreejs* [13] is a web-based tool that is difficult to use for those who have little knowledge about JavaScript or specific web programming visualization libraries. *HaploForge* [22] is another web application that is hard to use, as the meaning of the different symbols and connecting lines can be confusing for users who do not know about genetics; it is always difficult to know how to proceed to the next operation. *Ped_draw* [23] can be used only when users prepare pedigree data first. In addition, many tools such as *panogram* [16] and *HaploPainter* [17] are not free to use for all users. There are also some tools such as *PediDraw* [24] and *Madeline 2.0 PDE* [25] that were first released over a decade ago, and access to them is presently unstable or even disabled.

iPed is a mobile-based and self-service tool that can be used easily in place of software needing installation or web-based tools requiring professional knowledge. Collecting pedigree data is easier, as users only need to answer some questions asked by an intelligent robot and tap on some options. It is a simple and efficient tool for both professional and nonprofessional uses. Meanwhile, iPed provides a database to save all information submitted by users, providing greater convenience for subsequent uses. Additionally, iPed is free for everyone to use.

### Strengths and Limitations

iPed enables the collection of pedigree data through dialogue with an intelligent robot. As the pedigree data are usually complex, the possibility of missing some information is high, and that can lead to wrong conclusions. The dialogue with iPed helps the user recall their pedigree data more comprehensively. Questions asked by iPed include the following: “How many uncles do you have?” “Is your uncle married?” “How many children does your uncle have?” and “Do your uncle’s children have the phenotype?” Without any other operations, the users can answer the questions just by tapping on the “yes,” “no,” or “unknown” options, and the pedigree data collection is accomplished when the dialogue ends. The pedigree data visualization will then generate automatically. Moreover, considering that there are associations between diverse phenotypes, iPed will save all the information submitted by the users after the first use. Therefore, it is easy to look over the phenotypes that have been entered before. iPed offers substantial help in clinical diagnosis of complications and genetics studies about the correlations of different phenotypes.

Although iPed provides many novel functions, there are still some limitations. First, in the visualization of pedigree data, the color black traditionally indicates a person with the phenotype and white indicates one without the phenotype. iPed will offer a more powerful function if more colors are introduced, indicating different meanings. Second, users should select a phenotype before the pedigree data collection, so that the resultant picture shows information for the specified phenotype. How to present multiple phenotypes in a single picture will be considered in the future. We will constantly update and upgrade iPed to offer a significantly better user experience.

### Conclusions

iPed was developed as a mobile-based and self-service tool to collect and visualize pedigree data [26]; it can be used by professional researchers, clinicians, and those who possess little relevant knowledge. iPed shortens the amount of time patients spend in the hospital and improves the efficiency of the clinicians. With iPed, collection, management, and use of pedigree data will no longer be difficult.
